# Supplementary material for: High-Resolution Melting (HRM) Analysis for Rapid Molecular Identification of Sparidae Species in the Greek Fish Market
Source: Genes (Basel). 2023 Jun 12;14(6):1255. doi: 10.3390/genes14061255 (PMC10298334; doi:10.3390/genes14061255)
Supplement: Supplementary file 1 [file genes-14-01255-s001.zip › genes-2414091-supplementary.pdf]

genes

MDPI

Supplementary Materials:

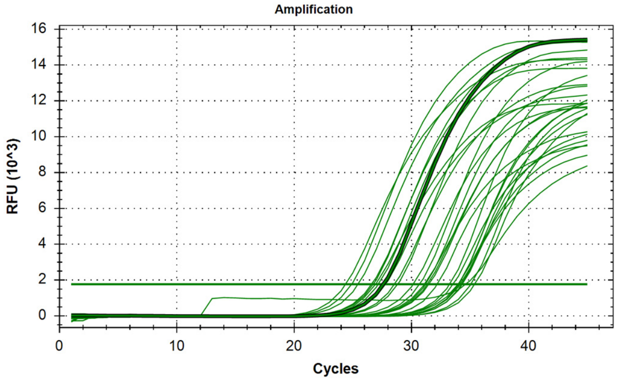

(**a**)

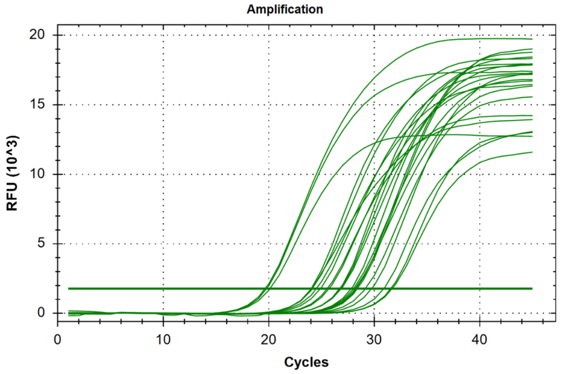

(**b**)

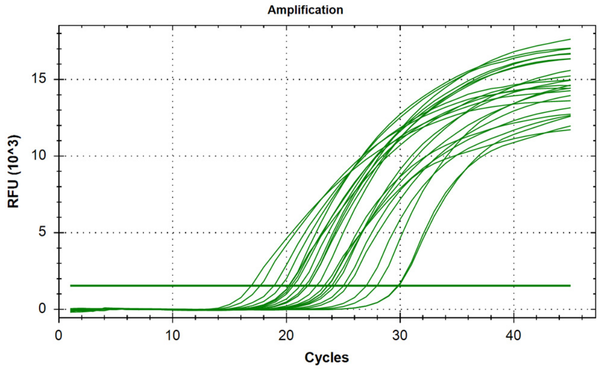

(**c**)

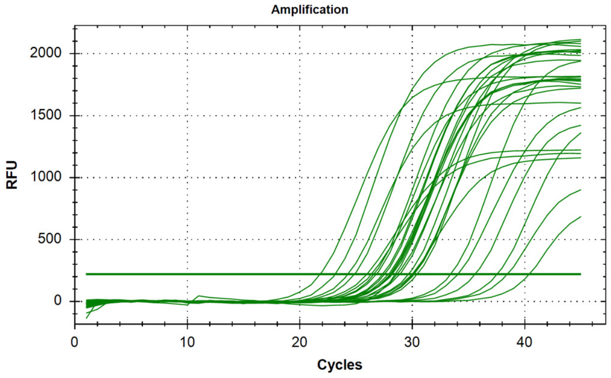

(**d**)

Figure S1. qPCR amplification curves of the samples tested with HRM as: (**a**) *cytb* for *P.pagrus* (**b**) *16s* for *P.pagrus* (**c**) *COI* for *D.dentex* (**d**) *16s* for *D.dentex*.

Table S1. Mean Cq values and SD for the examined species in HRM analysis for template concentration 10 ng.

Species	Cq±SD *COI*	Cq±SD *cytb*	Cq±SD *16s*
Pagrus pagrus	25,5±4,36	25,1±3,53	25,8±2,66
Pagrus major	31,3 ±2,71	32,9±1,79	26,5±4,43
Pagrus caeruleostictus	25,7±5,1	33,3±5,49	27,8±2,58
Dentex dentex	21,5±2,8	31,3±4,67	25,7±3,23
Dentex gibbosus	27,3±2,72	32,9±1,22	27,8±3,45
Dentex angolensis	35,6±2,45	34,5±1,49	28,4±6,22
Pagellus erythrinus	32,5±3,14	27,6±1,88	32,0±3,60

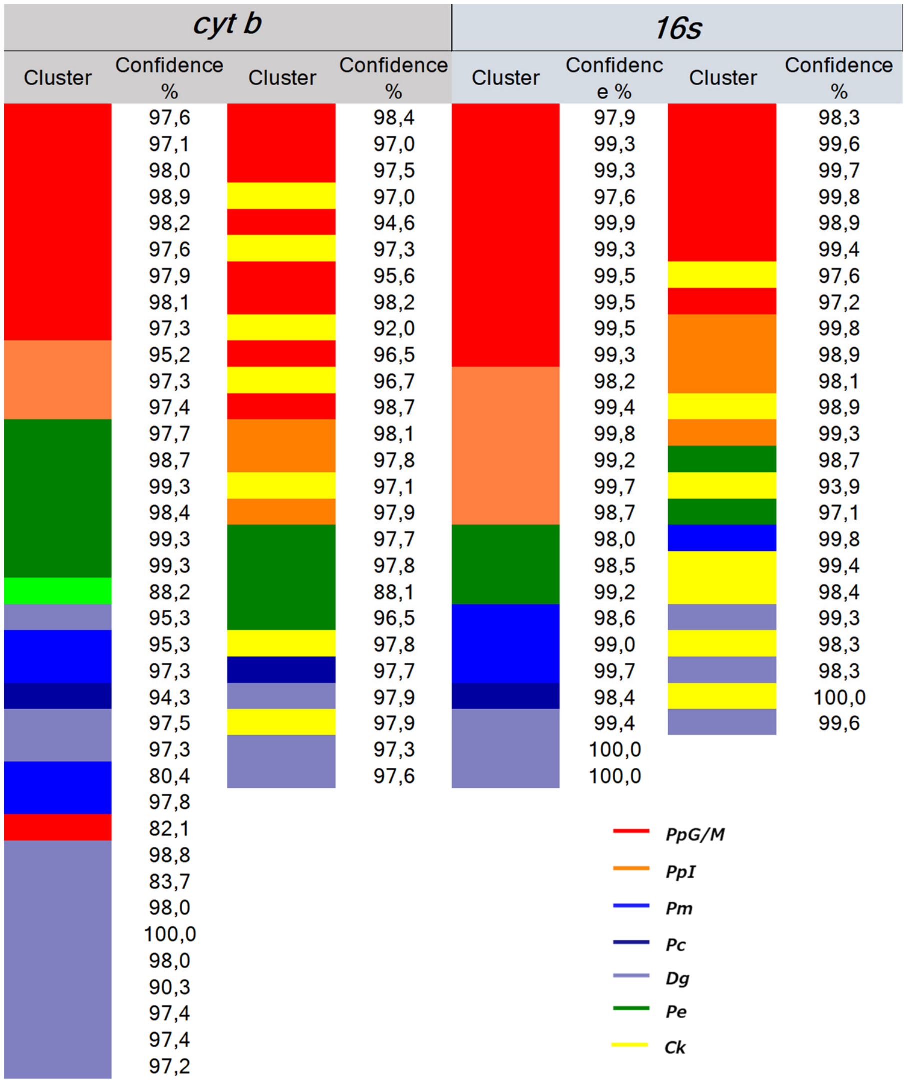
cyt b
16s
Cluster
Confidence %
Cluster
Confidence %
Cluster
Confidenc e %
Cluster
Confidence %
97,6 97,1 98,0 98,9 98,2 97,6 97,9 98,1 97,3 95,2 97,3 97,4 97,7 98,7 99,3 98,4 99,3 99,3 88,2 95,3 95,3 97,3 94,3 97,5 97,3 80,4 97,8 82,1 98,8 83,7 98,0 100,0 98,0 90,3 97,4 97,4 97,2
98,4 97,0 97,5 97,0 94,6 97,3 95,6 98,2 92,0 96,5 96,7 98,7 98,1 97,8 97,1 97,9 97,7 97,8 88,1 96,5 97,8 97,7 97,9 97,9 97,3 97,6
97,9 99,3 99,3 97,6 99,9 99,3 99,5 99,5 99,5 99,3 98,2 99,4 99,8 99,2 99,7 98,7 98,0 98,5 99,2 98,6 99,0 99,7 98,4 99,4 100,0 100,0
98,3 99,6 99,7 99,8 98,9 99,4 97,6 97,2 99,8 98,9 98,1 98,9 99,3 98,7 93,9 97,1 99,8 99,4 98,4 99,3 98,3 98,3 100,0 99,6
PpG/M
PpI
Pm
Pc
Dg
Pe
Ck

(a)

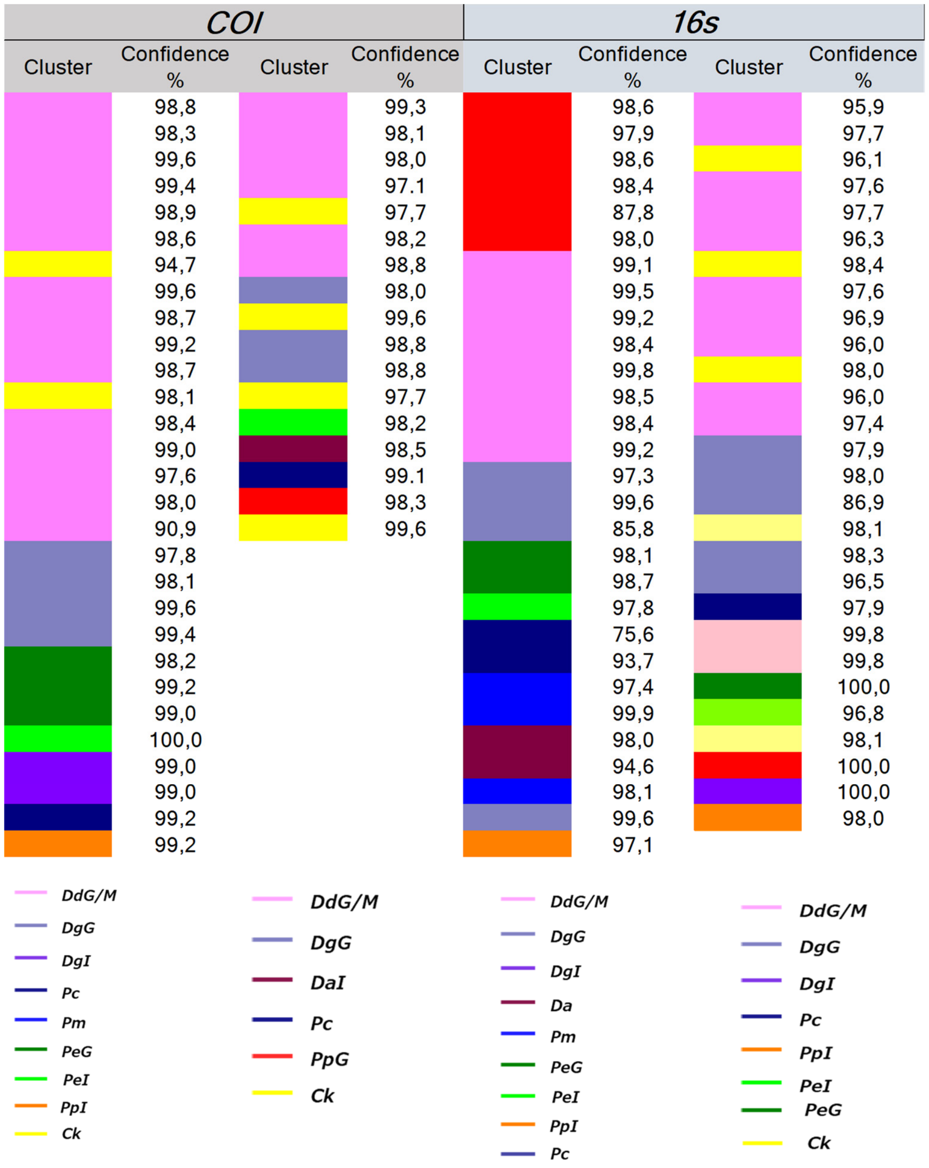

(**b**)

Figure S2. Maximum Confidence Percent values indicating the relative probability of a sample being grouped in a cluster. Melt curve shape sensitivity and the Tm difference threshold values are given in 3.3.3 for each fragment: (**a**) for genes examined in HRM for *P.pagrus* (**b**) for genes examined in HRM *for D. dentex.*

	PpG	PpI	Pm	Pc	Pe	Dg
PpG		96.460%	82.301%	88.496%	83.628%	87.168%
PpI	96.460%		80.531%	86.726%	80.973%	88.938%
Pm	82.301%	80.531%		78.761%	80.973%	82.743%
Pc	88.496%	86.726%	78.761%		79.204%	81.858%
Pe	83.628%	80.973%	80.973%	79.204%		86.283%
Dg	87.168%	88.938%	82.743%	81.858%	86.283%	

(a)

	PpG	PpI	Pm	Pc	Pe	Dg
PpG		98.710%	94.839%	94.872%	95.484%	94.516%
PpI	98.710%		94.839%	94.231%	95.484%	93.871%
Pm	94.839%	94.839%		94.872%	92.258%	94.516%
Pc	94.872%	94.231%	94.872%		92.308%	95.192%
Pe	95.484%	95.484%	92.258%	92.308%		94.516%
Dg	94.516%	93.871%	94.516%	95.192%	94.516%	

(b)

Figure S3. Percentage of sequence identity for the fragments used in HRM analysis of *P. pagrus* identification (**a**) cytb; (**b**) *16s*.

	Dd	DaI	Dg1	Dg2	PeG1	PeG2	PeI	Pc	PpG1	PpG2	PpI
Dd		91.379%	91.379%	90.517%	92.241%	91.379%	92.241%	92.241%	90.517%	89.655%	91.379%
DaI	91.379%		89.655%	88.793%	87.069%	87.931%	88.793%	90.517%	90.517%	89.655%	91.379%
Dg1	91.379%	89.655%		99.138%	93.103%	93.966%	93.103%	96.552%	93.966%	93.103%	93.103%
Dg2	90.517%	88.793%	99.138%		92.241%	93.103%	92.241%	95.690%	93.103%	93.966%	92.241%
PeG1	92.241%	87.069%	93.103%	92.241%		99.138%	98.276%	93.103%	92.241%	91.379%	91.379%
PeG2	91.379%	87.931%	93.966%	93.103%	99.138%		99.138%	92.241%	91.379%	90.517%	90.517%
PeI	92.241%	88.793%	93.103%	92.241%	98.276%	99.138%		93.103%	90.517%	89.655%	91.379%
Pc	92.241%	90.517%	96.552%	95.690%	93.103%	92.241%	93.103%		94.828%	93.966%	95.690%
PpG1	90.517%	90.517%	93.966%	93.103%	92.241%	91.379%	90.517%	94.828%		99.138%	99.138%
PpG2	89.655%	89.655%	93.103%	93.966%	91.379%	90.517%	89.655%	93.966%	99.138%		98.276%
PpI	91.379%	91.379%	93.103%	92.241%	91.379%	90.517%	91.379%	95.690%	99.138%	98.276%	

(a)

	Dd	Da	Dg	Pm	Pe	PpG	PpI	Pc
Dd		90.385%	93.269%	90.385%	90.385%	91.667%	91.026%	94.231%
Da	90.385%		93.871%	94.839%	92.258%	92.258%	92.258%	93.590%
Dg	93.269%	93.871%		94.516%	94.516%	94.516%	93.871%	93.910%
Pm	90.385%	94.839%	94.516%		92.258%	94.839%	94.839%	93.590%
Pe	90.385%	92.258%	94.516%	92.258%		95.484%	95.484%	91.026%
PpG	91.667%	92.258%	94.516%	94.839%	95.484%		98.710%	93.590%
PpI	91.026%	92.258%	93.871%	94.839%	95.484%	98.710%		92.949%
Pc	94.231%	93.590%	93.910%	93.590%	91.026%	93.590%	92.949%	

(b)

Figure S4. Percentage of sequence identity for the fragments used in HRM analysis of *D. dentex* identification (**a**) *COI*; (**b**) *16s*.
